# Intensive vasodilatation in the sciatic pain area after dry needling

**DOI:** 10.1186/s12906-015-0587-6

**Published:** 2015-03-20

**Authors:** Elżbieta Skorupska, Michał Rychlik, Włodzimierz Samborski

**Affiliations:** Department of Rheumatology and Rehabilitation, Poznan University of Medical Sciences, Fredry 10, 61-701 Poznań, Poland; Department of Virtual Engineering, Poznan University of Technology, Plac Marii Skłodowskiej-Curie 5, 60-965 Poznań, Poland

**Keywords:** Dry needling, Vasodilatation, Myofascial pain, Sciatica

## Abstract

**Background:**

Short-term vasodilatation in the pain area after dry needling (DN) of active trigger points (TrPs) was recorded in several cases of sciatica. Moreover, the presence of TrPs in sciatica patients secondary to primary lesion was suggested. Still, it is not known how often they occur and if every TrPs can provoke vasomotor reactions.

The purpose of this study was to evaluate the prevalence of active TrPs among subacute sciatica patients and the response to DN under infrared thermovision (IRT) camera control.

**Method:**

Fifty consecutive Caucasian patients (mean age 41.2 ± 9.1y) with subacute sciatica were diagnosed towards gluteus minimus TrPs co-existence. Based on TrPs confirmation, patients were divided into two groups: TrPs-positive and TrPs-negative, than DN under IRT control was performed. Skin temperature changes and the percentage size of vasomotor reactions in the pain area were evaluated if present.

**Results:**

The prevalence of active TrPs was 32.0%. Every TrPs-positive presented vasodilatation dependent on TrPs co-diagnosis (r = 0.72 p < 0.000) and pain recognition during DN (r = 0.4 p < 0.05). The size of vasodilatation in TrPs-positive subjects was: post-DN 12.3 ± 4.0% and post-observation 22.1 ± 6.1% (both p = 0.000) versus TrPs-negative: post-DN 0.4 ± 0.3% and post-observation 0.4 ± 0.2%. A significant temperature increase in the thigh and calf was confirmed for TrPs-positive subjects only (both p < 0.05). Post-DN and post-observation temperatures were as follows: average (thigh:1.2 ± 0.2°C; 1.4 ± 0.2°C, both p < 0.05 and calf: 0.4 ± 0.2°C; 0.4 ± 0.3°C, both p < 0.05) and maximum (thigh 1.4 ± 0.3°C 1.6 ± 0.3°C; both p < 0.05).

**Conclusions:**

The presence of active TrPs within the gluteus minimus muscle among subacute sciatica subjects was confirmed. Every TrPs-positive sciatica patient presented DN related vasodilatation in the area of referred pain. The presence of vasodilatation suggests the involvement of sympathetic nerve activity in myofascial pain pathomechanism. Although the clinical meaning of TrPs in subacute sciatica patients is possible, further studies on a bigger group of patients are still required. Trial registration: Australian New Zealand Clinical Trials Registry ACTRN12614001060639. Registered 3 October 2014.

## Background

A growing interest in myofascial pain syndrome caused by trigger points is currently observed among researchers. Myofascial pain syndrome is a type of muscle pain caused by trigger points, which are responsible for sensory, motor and autonomic symptoms. Trigger points (TrPs) defined as hyperirritable spots in the taut bands of muscle fibers can be classified as either active or latent. While an active TrP is characterized by spontaneous pain or pain in response to movement, stretch or compression, a latent TrPs is a sensitive spot with pain or discomfort in response to compression only. Additionally, appropriate stimulation of TrPs can elicit spinal cord reflex called “twitch response”, which is characteristic of TrPs exclusively [1-3]. Trigger points appear to be located in the vicinity of the endplate zone, and it has been hypothesized that there are multiple TrP loci which contain a sensory component (irritated nociceptor) and a motor component (dysfunctional end plate) [4]. However, there is no overlap between trigger points and the innervation zone [5]. Sustained contractures of taut bands cause local ischemia and hypoxia in the core of TrPs [6], provoking low oxygen levels which may lead to a significant drop in pH. A low pH level in TrPs, e.g. below 5, can easily excite muscle nociceptors and nociceptive input can induce neuroplastic changes in the spinal dorsal horn and likely in the brainstem [7,8]. Existing research suggests that sympathetic-sensory interaction within TrPs can contribute to local and referred pain [9]. Additionally, the presence of autonomic phenomena, e.g. vasomotor, pilomotor, etc. in the area of referred pain was postulated by Travell and Simons [1]. However, it is believed that the presence of autonomic phenomena in the area of referred pain is limited to severe active trigger points exclusively. Some authors confirmed the presence of discrete vasoconstriction after noxious stimulation of latent trigger points [10,11] and suggested the use of laser Doppler flowmetry rather than the less sensitive infrared thermovision (IRT) camera. Interestingly, opposite to this point of view, some evidence of intensive short-term vasodilatation corresponding to the pain area of sciatic and sciatic-type leg pain after dry needling of active TrPs within the gluteus minimus muscle under IRT control was proved [12,13]. Although the exact mechanism of dry needling remains unknown, it is believed that treatment by dry needling (fast, repetitive insertion of a thin needle into a TrP) can stop intensive nociceptive input and reverse neuroplastic changes provoked by trigger points.

Short-term vasodilatation after dry needling of active TrPs was recorded in several cases and it is unknown whether noxious stimulation of every active trigger point can provoke vasomotor reactions. Moreover, the presence of trigger points in sciatica patients secondary to a primary lesion, e.g. a vertebral disc lesion, was suggested [14]. Still, it is unknown how often they occur. The occurrence of TrPs within several muscles is indicated as a possible source of “sciatic pain”. The gluteus minimus muscle together with quadratus lumborum and sometimes piriformis, peroneus longus, etc. play a significant role here [1].

Because the prevalence of TrPs among sciatica patients is unknown, the first aim of this study was to evaluate the prevalence of active gluteus minimus trigger points among subacute sciatica patients. The second aim was to check the hypothesis that dry needling of gluteus minimus TrPs can provoke autonomic response in the area where trigger points refer pain (autonomic referred pain – AuRP).

## Methods

### Ethics statement

**Figure 1:**
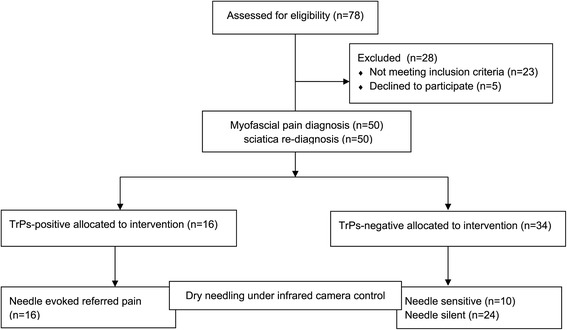
**A flow diagram of the study design.**

The study was conducted in accordance with the Declaration of Helsinki approved by the Ethics Committee of Poznan University of Medical Sciences (no. 630/13). All subjects gave written informed consent to participate in the study before data collection. A detailed description of all examination and treatment procedures, including dry needling (DN), and risks involved in this study, was provided to the participants. Participants had the right to refuse DN treatment and withdraw from the study at any time without penalty. A flow diagram of the study design was shown as Figure 1.

### Subjects

Seventy eight Caucasian patients (both sexes) with subacute sciatica were enrolled to the study, twenty three of them did not meet inclusion criteria and five refused to participate in the study because of the needle fear. Finally, fifty Caucasian patients (men 19, female 31) with subacute sciatica (mean age 41.2 ± 9.1y) were recruited to the study consecutively from the University Pain Clinic. All participants were re-diagnosed with sciatica of radicular origin by an independent university neurologist based on clinical bedside examination, extensive neurological screening examination, accompanied by a positive straight leg test and magnetic resonance imaging results. Key inclusion criteria were as follows: diagnosis of sciatica, age between 30 and 60 (inclusive), both lower limbs present, pain duration 1–3 months, >3 on the 1–10 point VAS scale of leg pain, with this being the dominant pain problem. Patients were excluded from the study for a number of reasons, i.e. complex regional pain syndrome, cauda equina syndrome, previous back surgery, spinal tumors, scoliosis, pregnancy, coagulant treatment, disseminated intravascular coagulation, diabetes, epilepsy, infection, inflammatory rheumatologic diseases, stroke, or oncological history.

### Methods

#### Trigger points confirmation

In one week maximum, post neurological examination of all the sciatica subjects was conducted by an experienced specialist for myofascial pain syndrome co-existence – gluteus minimus active trigger points presence based on Travell and Simons’ diagnostic criteria [1]. After trigger points confirmation, patients were divided into two groups: TrPs-positive and TrPs-negative (control).

Active trigger points (TrPs) were confirmed if spot tenderness, recognition of pain and digitally evoked referred pain pattern of the gluteus minimus were present. The localization of the two most active TrPs was marked. For the non-TrPs subjects, the two most tender points were marked. According to Travell and Simons, the referred pain pattern of the gluteus minimus muscle (confirmatory sign) is rarely present under digital pressure of TrPs and it is usually evoked when the needle encounters TrPs [1]. Due to this fact, non-TrPs subjects who reported the two most important clinical features of TrPs after needle infiltration during the procedure (i.e. recognition of pain and referred pain pattern of the gluteus minimus muscle) were additionally analyzed as a likely TrPs subgroup.

#### Dry needling procedure under IRT control

Just after trigger points confirmation, every subject received the dry needling procedure under IRT control in the same University IRT laboratory. Dry needling (DN) in the present study is meant to be a potential diagnostic tool. However, DN performed for the purposes of the study may have a potentially therapeutic effect (pain relief), which was not evaluated.

##### Side-to-side IRT comparison

All of the patients were asked to define the pain level on the visual analogue scale and to draw their current pain pattern on a diagram. IRT side-to-side comparison of the painful area and the opposite extremity was performed in the standing position.

##### Thermovision camera application

Thermographic images were recorded by an expert following a standard protocol recommended by the Academy of Neuro-Muscular Thermography [15]. The expert also evaluated the images. Patients were instructed to avoid physiotherapy and manual therapy 24 hours prior to the test, and avoid using nasal decongestants, analgesics, anti-inflammatory drugs or any substances affecting the sympathetic function. They were also instructed not to drink coffee or alcohol and refrain from smoking 2 hours before the recording.

A thermovision touchless camera (NEC-AVIO TVS-200EX) using a 8-14 μm wave band, temperature resolution better than 0.080°C, sensitivity 80 mK and working in real time was applied. The camera was equipped with a high-speed (60 Hz) uncooled FPA 320x240 (HxV) pixels VOx (vanadium oxide) microbolometer. For data analysis (thermal images), the specialist program “Thermography Studio 2007 Professional” was used.

To obtain the stability of patient body temperature and to ensure the adjustment of the recording camera’s temperature to the interior conditions, the evaluation began 30 minutes after the patient had entered the examination room. Thermal isolation of the evaluated area from other thermal factors that might have influenced the evaluation, including other parts of the patient’s and doctor’s bodies, was ensured. Moreover, when performing thermovision imaging, the general rules of camera usage were followed.

##### Protocol of dry needling/infrared thermography control

The patient’s position for needling of the gluteus minimus muscle was the side lying position. The muscle was needled with flat palpation perpendicular to the muscle along the counter of the iliac crest. Strong depression of the subcutaneous tissue was applied in order to reduce the distance of the skin from the muscle. Depth of penetration depended on the amount of adipose tissue [16]. Therapeutic needling was performed with 0.30 mm diameter, 60 mm long sterile acupuncture needles SE L (Serin Corp, Shizuoka, Japan). Each needle was packed separately. The area to be observed by IRT was chosen according to the gluteus minimus referred pain pattern. The examined patients were positioned on the side, on the uninvolved extremity with the hip and knee flexed. In this position, thermovision images of the patient were recorded. For adequate representation of dimensions, a calibration standard was applied.

Then, all subjects received a dry needling (DN) session under IRT. The time of needling was 5 minutes for every given point. During the whole procedure, the subarea of referred pain reported by the patient (thigh, calf, foot) was recorded. After the needling of both marked points was completed, further thermovision imaging was performed. The IRT observation lasted for six consecutive minutes post-DN.

The analysis of thermograms assumed skin temperature (T_sk_) changes (maximum, minimum, average) in the observed area after the dry needling session during 6 minutes post-DN. Additional analysis assumed the calculation (in cm^2^) of skin temperature (T_sk_) changes above the maximum (vasodilatation) or under the minimum (vasoconstrictions) temperature. The expected vasomotor changes in the area of TrPs related referred pain were named autonomic referred pain (AuRP) if present. The size of each AuRP was recalculated from cm^2^ to the percentage value.

### Statistical analysis

According to William Cochran’s assumption for X^2^ test, to conduct the test the quantity of n = 5 is required. It was assumed that in the examined group three subgroups were present, namely: sciatica with trigger points co-existence; sciatica without trigger points co-existence (needle silent); sciatica without trigger points co-existence (needle sensation). Additionally, the subgroups were analyzed based on gender.

As a result, the information about the minimal size of the sample was obtained (3 features * 2 gender categories* the quantity of n = 5 per subgroup; n = 30). However, for the strong evidence of data the examined was n = 50 because the prevalence of trigger point among sciatica patients is unknown. For the strong evidence of data presented, the significance level was set based on the exact tests, not on the default asymptotic method. Exact two-way Mann–Whitney U tests were performed in order to ensure that data are representative of the whole population of possible data values. Tests were applied to compare the differences for maximum, minimum and average skin temperature and the percentage size of expected autonomic phenomena for the state after dry needling and secondly for the post-DN state. Pearson correlation with two-tailed significance test was applied to define the dependency of the autonomic phenomenon occurrence. All comparisons were completed, with trigger points co-existence being the differentiating criterion.

Additionally, the two-way Jonckheere-Terpstra test with the same terms was conducted in order to observe the tendency of skin temperature changes and autonomic phenomena occurrence dependent on TrPs, likely TrPs and non-TrPs presence.

Values, figures and tables in the text are expressed as ± standard error of the mean (SEs). Significance level was set at p < 0.05. Statistical analysis was performed using IBM SPSS Statistics, version 20".

## Results

Based on IRT, side-to-side (painful to non-painful leg) average skin temperature (T_avr_) comparison before the dry needling (DN) session revealed ΔT_avr_ of 0.01°C.

**Figure 2:**
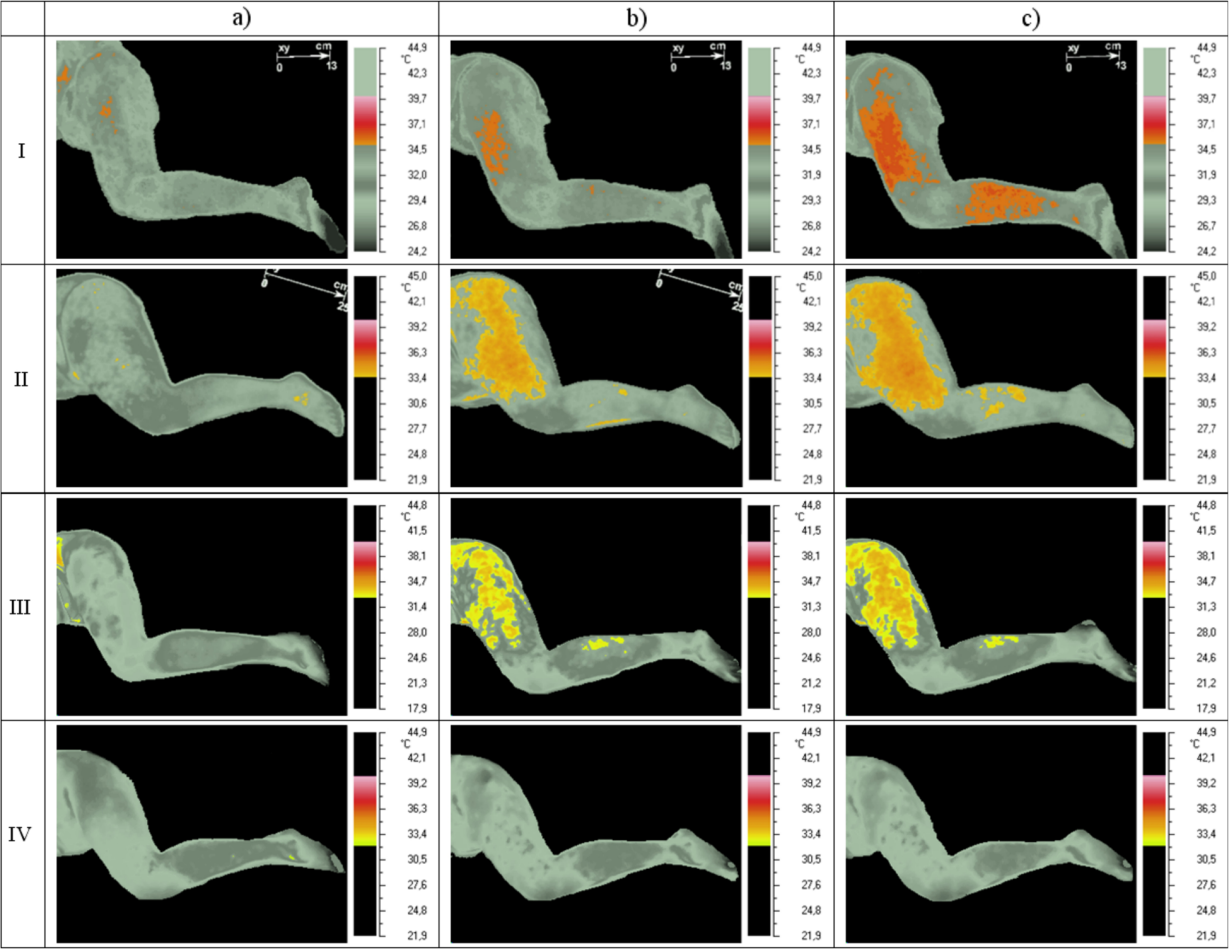
**Thermogram visualizing the area of maximum temperature above the initial state (AuRP) dependent on TrPs co-existence.** Legend: In order to describe the area of AuRP using IRT, patients’ temperature reaction was isolated (grey pictures). TrPs-positive cases are presented in rows I-III, non-TrPs case in row IV. In columns **(a)-(c)**, pre-DN (initial) state, post-DN state, and post-observation state are presented, respectively. TrPs-positive cases (I-II) felt pain radiating to the calf and case III – to the foot. The thermograms presented short-term vasodilatation in the area where DN provoked pain during the procedure. Color differences (case I-III) reflected ranging skin temperature and have no relevant impact.

The prevalence of gluteus minimus active trigger points (TrPs) in the group of subjects was 32.0%. IRT evaluation confirmed short-term vasodilatation for all TrPs positive subjects (Figure 2). None of the subjects presented a skin temperature decrease (vasoconstriction).

Pearson correlation with two-tailed significance test confirmed that the presence of vasodilatation (Figure 2) was dependent on TrPs co-diagnosis (r = 0.72 @p < 0.000; in detail: thigh r = 0.72 p < 0.005; calf r = 0.44 p < 0.005) and recognition of gluteus minimus referred pain evoked during the dry needling/IRT session - AuRP (r = 0.4 p < 0.05). There was no relationship between the localization of vasodilatation (Figure 2) and the patient’s daily pain (thigh, calf, foot) reported before the procedure.

The two-way Mann Whitney U test confirmed a significant increase of AuRP in TrPs-positive subjects (post-DN and post-observation; both p = 0.000). The size of AuRP in TrPs-positive subjects was as follows: post-DN 12.3 ± 4.0% and post-observation 22.1 ± 6.1%. Among non-TrPs subjects, only several hot spots were confirmed (post-DN 0.4 ± 0.3% and post-observation 0.4 ± 0.2%). Figure 2 shows exemplary short-term vasodilatation (AuRP).

### Skin temperature changes

**Figure 3:**
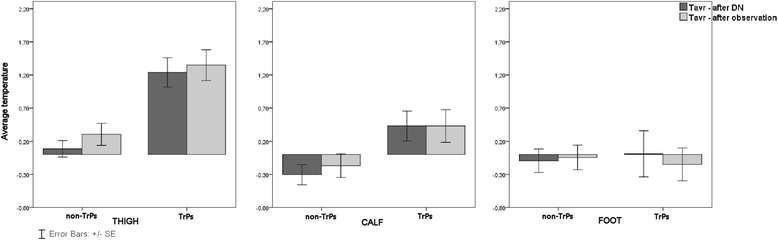
**Average skin temperature changes of the sciatic leg (thigh, calf and foot) dependent on trigger points confirmation.** Legend. The histograms reflected average temperature (T_avr_) changes depending on TrPs presence for the thigh, calf and foot in the light of two phases (post-dry needling and post-observation). A statistically significant T_avr_ increase of TrPs-positive sciatica corresponding to vasodilatation (thigh, calf) contrary to T_avr_ decrease (calf, foot) and insignificant T_avr_ thigh increase of non-TrPs sciatica subjects was confirmed.

**Figure 4:**
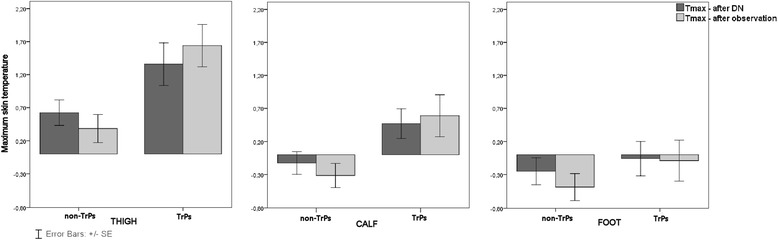
**Maximum skin temperature changes of the sciatic leg (thigh, calf and foot) dependent on trigger points confirmation.** A statistically significant T_max_ increase for the thigh and an insignificant increase for the calf of sciatica subjects manifesting TrPs co-existence reflected the presence of AuRP. For non-TrPs subjects, an insignificant increase was observed only for the thigh. For other parts of the leg of non-TrPs subjects and the foot of TrPs-positive subjects, T_max_ decrease was observed. Legend. The histograms reflected maximum temperature (T_max_) changes depending on TrPs presence for the thigh, calf and foot in the light two phases (post-dry needling and post- observation). A statistically significant T_max_ increase corresponding to vasodilatation (thigh, calf) contrary to T_avr_ decrease (calf, foot) and insignificant T_avr_ thigh increase of non-TrPs sciatica subjects was confirmed.

Dry needling provoked skin temperature changes for all subjects (n = 50) in all of the measured subareas, namely the thigh, calf and foot (Figures 3 and 4). However, a statistically significant T_sk_ increase in the thigh and calf was confirmed for TrPs-positive subjects only. The exact two-way Mann–Whitney U test showed that post-DN and post-observation there were significant increases in average temperature @(Figure 3) for the thigh (1.2 ± 0.2°C; 1.4 ± 0.2°C both @p < 0.05) and calf (0.4 ± 0.2°C; 0.4 ± 0.3°C both p < 0.05) for TrPs-positive subjects. Similarly, the exact two-way Mann–Whitney U test confirmed a significant increase in thigh T_max_ post-DN (1.4 ± 0.3°C) and post-observation (1.6 ± 0.3°C) (both p < 0.05) (Figure 4). Similar tendency for calf maximum temperature increase for TrPs-positive contrary to decrease for TrPs-negative subjects (both insignificant) was confirmed.

### Detailed analysis of non-TrPs subgroup

#### AuRP presence

Among non-TrPs subjects (n = 34), some of the patients @(n = 10) reported pain exacerbation following needle infiltration into the gluteus minimus muscle (likely TrPs). The presence of some hot spots above initial maximum temperature was confirmed. The size of AuRP for likely TrPs subjects was: post-DN 0.0 ± 0.0% and post-observation 0.1 ± 0.1%. The percentage size of AuRP area for needle negative non-TrPs subjects was: post-DN 0.6 ± 0.5% and post-observation 0.4 ± 0%.

#### Skin temperature changes

Dry needling provoked skin temperature changes for all non-TrPs subjects in all of the measured subareas, namely the thigh, calf and foot. However, no significant increases in average and maximum temperature were confirmed (Figure 3 and Figure 4).

### Data analysis of AuRP tendency dependent on TrPs, likely TrPs and non-TrPs presence

#### Post-DN state

The two-way Jonckheere-Terpstra test revealed a significant positive tendency for AuRP presence (p < 0.05; Z statistic value 2.3; effect size 0.4%) depending on TrPs presence. The mean value of AuRP percentage area was: 0.61 ± 0.45% for non-TrPs, 0.0 ± 0.0% for likely TrPs and 12.25 ± 3.97% for TrPs-positive subjects. Similarly, for skin temperature changes the two-way Jonckheere-Terpstra test revealed a significant positive tendency for: thigh maximum temperature increase (p < 0.05; Z statistic value 2.29; effect size 0.42%), thigh average temperature increase @(p < 0.000; Z statistic value 3.29; effect size 0.6%) and calf average temperature increase (p < 0.005; Z statistic value 2.53; effect size 0.46) dependent on trigger point presence.

#### Post-observation state

The two-way Jonckheere-Terpstra test revealed a significant positive tendency for AuRP presence (p < 0.000; Z statistic value 3.8; effect size 0.7%) depending on TrPs presence. The mean value of AuRP percentage area was: 0.44 ± 0.31% for non-TrPs, 0.12 ± 0.12% for likely TrPs and 22.07 ± 6.10% for TrPs-positive subjects. Similarly, for skin temperature changes the two-way Jonckheere-Terpstra test revealed a significant positive tendency for: thigh T_avr_ increase (p < 0.002; Z2.93; effect size 0.54) and calf T_avr_ increase (p < 0.002; Z statistic value 2.16; effect size 0.39) dependent on trigger point presence and needle reactions.

## Discussion

The present study demonstrated that active trigger points co-exist in around one in three subacute sciatica patients. This is the first study examining the significance of active trigger points in subacute sciatica patients. However, the co-existence of active trigger points in nonspecific low back pain and cervical radiculopathy was confirmed [17,18].

Nevertheless, the most important result of the present study is that dry needling under IRT control provoked a TrPs related significant temperature increase (p < 0.05) and revealed short-term vasodilatation corresponding to the subject’s pain distribution (Figure 2). Moreover, the presence of vasodilatation depended on the gluteus minimus referred pain evoked during the dry needling/IRT session (AuRP; @r = 0.72 p < 0.000) rather than the pain pattern of the daily complaint.

The presence of autonomic phenomena in the area of trigger point referred pain, e.g. vasomotor, pilomotor, etc., was considered an extremely rare state, limited to severe active TrPs only. The results of the present study are opposite. All subjects presented short-term vasodilatation when TrPs co-existed. Moreover, the AuRP covered on average around one-fifth of the lower extremities subarea (Figure 2). Such expansive vasodilatation, far away from the needling point, is unusual compared to other studies. There are only two case studies confirming similar results of TrPs needling, but the rest of available data indicate no vessel reactions, vasoconstriction or flare/vasodilatation 10 cm from the needled acupuncture point in healthy subjects and patients [10-13,19,20].

Additionally, IRT side-to-side comparison of subjects before the procedure indicated the norm and there was no difference between TrPs-positive and -negative subjects. This can explain why previously the usefulness of IRT was controversial [1,21].

Moreover, the results of the present study are opposite to those of Zhang et al. and Kimura et al., who presented latent TrPs related vasoconstriction after nociceptive stimulation under laser Doppler flowmetry control rather than IRT detectable skin temperature changes [10,11]. IRT observation of pain patients after noxious stimulation is the new way of IRT evaluation apart from side-to-side comparison established as the most valid [22]. However, some relevance of IRT for trigger point referred pain evaluation has lately been proved [23]. The idea of IRT observation of referred pain by Haddad et al. [23] was an appropriate direction in the light of the present study. However, the choice of the muscle and data analysis is not compelling. The referred pain pattern of the masseter is not as extensive as that of the gluteus minimus and is limited to several small subareas on the face and teeth only. Additionally, face temperature can be affected by psychological stress. Finally, the analysis of one single thermogram is not sufficient [23].

Another important aspect is that every TrPs-positive sciatica subject developed short-term vasodilatation, which indicates the involvement of the sympathetic nervous system (SNS) in trigger point pain propagation, which was suggested before [9]. Additionally, the results of the present study support Simons’ integrated hypothesis of TrPs etiology, in which the autonomic modulation was thought to have a potential influence on the increase of ACh release, which can aggravate symptoms caused by TrPs [24]. Moreover, the involvement of the SNS among sciatica patients diagnosed with neuropathic pain co-existence was proved in more than 30% of subjects [25]. Despite the occurrence of vasodilatation, it is not known how important the SNS involvement in pain propagation is among sciatica patients manifesting TrPs co-existence. However, it seems that dry needling can be a promising tool for treatment-resistant sciatica patients with coexisting TrPs because of postulated enhanced motor unit activity and facilitation of muscle pain at TrPs after maneuvers increasing sympathetic outflow [26].

### Analysis of non-TrPs subjects presenting needle evoked referred pain (likely TrPs)

Taut band presence and pain recognition are the two most important clinical criteria of trigger point diagnosis, and referred pain is a confirmatory sign. Due to the anatomy of the gluteus minimus muscle, the taut band is mostly not accessible but TrP spot tenderness can be clearly localized. However, it was reported that the referred pain pattern can be induced by the needle encountering TrP rather than by sustained pressure [1]. The positive tendency for AuRP presence dependent on TrP (p < 0.000) may indicate the importance of referred pain after digital pressure during TrPs diagnosis. Additionally, it seems that AuRP presence supported by a significant Tsk increase rather than maximum and average temperature measures only should be suggested for objective IRT confirmation of TrPs. Moreover, in the light of AuRP presence in the likely TrPs subgroup it seems that diagnosis of TrPs for needle reactive gluteus minimus muscle can probably not be clinically important.

### Detailed analysis of short-term vasodilatation presence

The results of the present study indicate the biphasic nature of AuRP. The dry needling session was followed by a further increase in skin temperature and spreading of vasodilatation (Figure 2). The exact mechanisms of cutaneous active vasodilator system functions as well as the dry needling mechanism remain enigmatic [27]. Additionally, the translation of mechanical stimuli into biochemical information is poorly understood. Kellogg [27] observed biphasic vasodilatation because of local warming with an initial increase mediated by an axon reflex followed by a plateau phase which requires the generation of nitric oxide (NO) by nitric oxide synthases. Interestingly, apart from the axon reflex contribution (substance P or calcitonin gene related peptide) also the importance of NO in vasodilatation mechanism related to intramuscular acupuncture was suggested [28].

The theories put forward by Lewis and Hong [4,29] can give some interesting explanation of AuRP presence described in the present study. Lewis formed the hypothesis that injury of sensory fibers provokes antidromic propagation of action potentials to collateral nerve fibers which liberate a chemical substance causing for flare and enhanced sensitivity of other sensory axons responsible for pain. Interestingly, blocking central nerve trunks [30] or cutting nerves [29] does not block the flare, which suggests that the central nervous system must receive some decisive input from the nerve fibers of the region exposed to the stimulus and the flare is a consequence of neural factors that operate peripherally. It was proposed that chemospecific peripheral nerve fibers may sensitize low- and high-threshold mechanoreceptive interneurons in the dorsal horn [31]. However, that theory is suitable for flare/vasodilatation in around a 6 cm area from the injury/needling, not expansive vasodilatation (AuRP) showed in the present study. Hong [4] suggests that all trigger point phenomena, such as referred pain, autonomic phenomena, twitch response and motor dysfunction, are dependent on the spinal cord reflex via “neural circuits” in the spinal cord. In the present study, the localization of AuRP was related to the referred pain provoked during dry needling, not the patient daily pain. Skorupska et al. observed the same vessel reactions and suggested that the extrication of twitch response and referred pain during dry needling via Hong’s “neural circuits” can explain the unusual presence of AuRP in the pain area [4,12,13]. Moreover, in addition to vasomotor responses, the area of referred pain from TrPs is also the typical site of sensory changes (hyperalgesia), which have been also attributed to possible sympathetic mechanisms (especially at superficial level) [32]. Further studies considering post-dry needling vasodilatation analysis are required.

The presence of dry needling related vasodilatation in all TrPs is quite promising with respect to the possibility of using IRT as an objective TrPs confirmation tool. However, it is important to check if all TrPs within muscles can develop vasodilatation. Additionally, the presence of AuRP indicate the involvement of the sympathetic nervous system in pain propagation in sciatica patients. Nevertheless, it is not known how clinically important TrPs presence in this group of patients is. Further studies are still required.

### Limitations of the study

The significant prevalence of TrPs among subacute sciatica subjects was confirmed. Although observed vasodilatation was characteristic of all TrPs-positive subjects, for strong methodological evidence a cross-over study is required. Assessment of DN influence on healthy subjects and IRT observation without DN are needed. Additionally, female fertile phase and its possible impact for the autonomic response should be observed in the future.

## Conclusions

The presence of active TrPs within the gluteus minimus muscle among subacute sciatica subjects was confirmed. All TrPs-positive sciatica patients presented DN related vasodilatation in the area of referred pain. Moreover, the presence of TrPs related autonomic phenomenon suggests the involvement of sympathetic nerve activity in myofascial pain pathomechanism. Although the clinical meaning of TrPs in subacute sciatica patients is possible, further studies on a bigger group of patients are still required.

## Abbreviations

AuRP: Autonomic referred pain
DN: Dry needling
IRT: Infrared thermovision camera
NO: Nitric oxide
SNS: Sympathetic nervous system
T_avr_: Average temperature
T_max_: Maximum temperature
T_sk_: Skin temperature
TrPs: Trigger points
VAS: Visual analogue scale
